# Solitary breast neurofibroma: imaging aspects

**DOI:** 10.3332/ecancer.2018.800

**Published:** 2018-01-23

**Authors:** Anna Rotili, Federica De Maria, Benedetta Di Venosa, Mariacristina Ghioni, Maria Pizzamiglio, Enrico Cassano, Michela Moratti

**Affiliations:** 1 Division of Breast Radiology, European Institute of Oncology, Milan 20137, Italy; 2 Division of Pathology, European Institute of Oncology, Milan 20137, Italy; 3 Ospedale Santa Maria delle Croci, Centro di Prevenzione Oncologica, Viale Randi 5, Ravenna 48121, Italy

**Keywords:** breast disease, neurofibroma, magnetic resonance

## Abstract

Neurofibromas are benign peripheral nerve sheath tumours, which are usually solitary and sporadic. Solitary neurofibromas of the breast are rare. The most common location of a breast neurofibroma is the nipple–areola complex.

We report a rare case of a 56-year-old woman with a solitary neurofibroma of the right breast sulcus.

## Introduction

Neurofibromas are benign nerve sheath tumours composed of Schwann cells, fibroblast and collagen fibers [1]. Localised neurofibromas are the most common form of neurofibroma (about 90%) [2]. Neurofibromas are very rarely found in the breast [3]. When not associated with neurofibromatosis type 1 (NF-1), neurofibromas might rarely undergo malignant transformation [4]. However, accurate diagnosis is important given the morbidity and psychological distress associated with such tumours.

## Report

**Figure 1.**
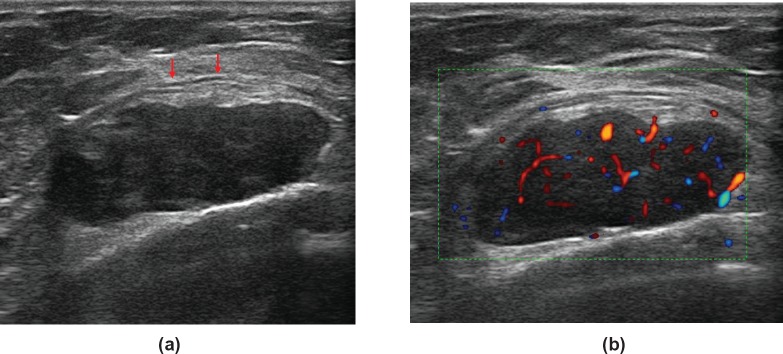
A 56-year-old woman with neurofibroma of the right sulcus: (a) US reveals a solid mass, oval-shaped, with circumscribed margins; the mass abuts the chest wall without signs of infiltrations (arrows). (b) Colour-Doppler shows high vascularity.

A 56-year-old woman with no family/personal history of breast cancer was presented with palpable nodularity in the right breast sulcus. An ultrasound (US) examination conducted at our hospital revealed a highly vascular hypoechoic round lesion of 3 cm, with smooth well-defined margins, deeply seated under the muscular aponeurotic fascia (Figure 1). The most recent mammography did not show any pathological findings.

**Figure 2.**
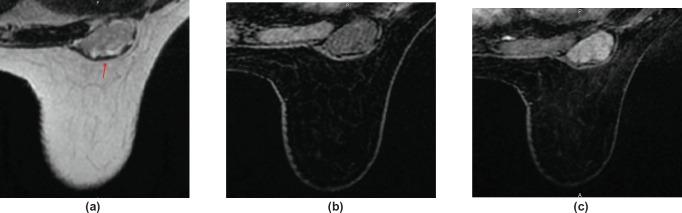
A 56-year-old woman with neurofibroma of the right sulcus: (a) T2W axial MR images demonstrate the mass is heterogeneously hyperintense compared to muscles and hypointense compared to fat tissues. The mass is within the breast but seated under the muscular aponeurotic fascia (arrow). (b) and (c) Axial T1W images with fat suppression; before gadolinium injection, the mass appears hypointense, with well-defined margins; (c) after gadolinium injection, the mass shows a heterogeneous enhancement.

A clinical evaluation was carried out after which the surgeon requested a contrast-enhanced magnetic resonance (MR) of the breast to better assess the lesions and to evaluate its relationship with the pectoralis muscle. Dynamic contrast-enhanced MR showed a 3-cm well-defined, encapsulated lesion of the right breast sulcus abutting the chest wall. The lesion was surrounded anteriorly by glandular and fatty breast tissues. The lesion was characterised by low T1 signal intensity and heterogeneously high T2 signal intensity. Contrast enhancement was highly heterogeneous (Figure 2). Diffusion-weighted (DW) images and related apparent diffusion coefficients did not show signal restriction.

**Figure 3.**
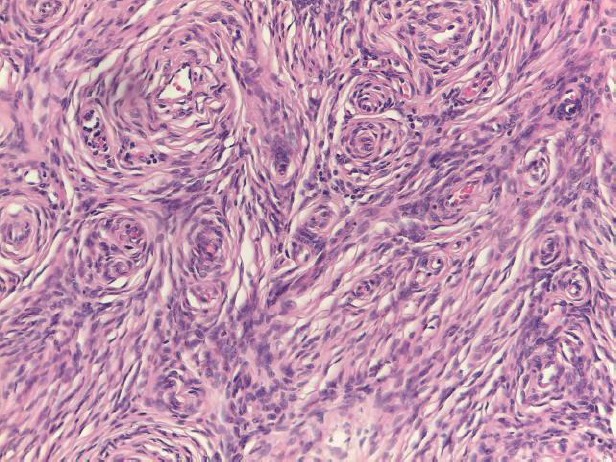
Spindle cell lesion composed of slender fibroblast-like cells with storiform pattern and very low amount of stroma.

**Figure 4.**
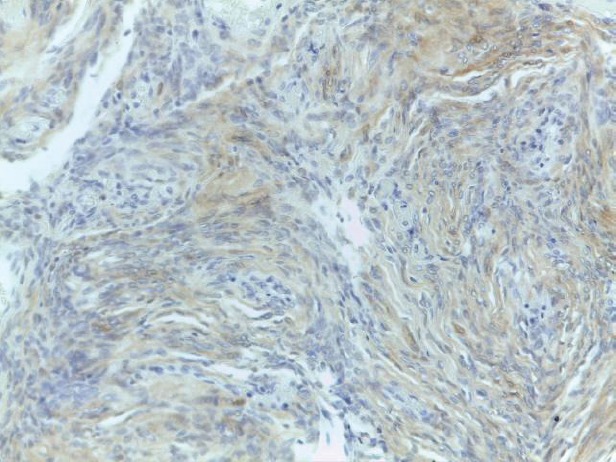
Focal and weak immunoreactivity for S-100 protein.

In the light of the morphological characteristics of this lesion, the enhancement pattern and the sudden onset, a core biopsy was requested and a subsequent excision was performed. Histopathologic analysis of the mass revealed a spindle cell lesion composed of fibroblast-like cells arranged in storiform pattern with little stroma, no features of malignancy and no mitoses (Figure 3). Immunohistochemistry revealed the positivity of the neoplastic cells for p75 and S-100 protein (Figure 4), markers of neural origin, and a negativity for markers of vascular, muscular and epithelial origin such as CD31, CD34, desmin, smooth muscle actin and cytokeratins. A diagnosis of neurofibroma was established.

## Discussion

Neurofibromas are benign slow growing tumours of the peripheral nerve sheath [1]. Peak presentation is between 20 and 30 years of age. There is no sex predilection [5].

The clinical presentation is that of a tissue solitary mass within the dermis or in the subcutis. Three different types of neurofibromas have been described (localised, diffuse and plexiform). The localised variety is the most common, representing approximately 90% of these lesions, and the vast majority are solitary and not associated with NF [2]. Breast involvement is rare, the most common location in the breast being the nipple–areola complex [3]. The radiographic features of neurofibroma depend on the histology of the lesion.

The US examination shows a well-defined round-shaped lesion; the lesion can either be hypoehoic (simulating a fibroadenoma) or anechoic with posterior acoustic enhancement (simulating a simple cyst) [6]. CT examination shows a well-defined, hypodense mass with minimal or no contrast enhancement [7]. MRI examination shows low to intermediate signal on T1-weighted (T1W)images and heterogeneously high signal on T2-weighted (T2W) images. The high T2 signal intensity is due to areas of cystic degeneration or to presence of myxoid matrix. Low T2 signal intensity is given by the presence of collagen and fibrous tissue. Areas of low T2 signal are those that enhance after gadolinium administration and lead to heterogeneous pattern [8, 9].

The US examination performed prior to the core-biopsy showed an oval-shaped lobulated mass of the right anterior chest wall, hypoechoic, with well-defined margins. The mass was deeply seated under the aponeurotic fascia without signs of perinodular infiltration. Colour-Doppler examination showed high vascularity (Figure 1).

At breast MR, the mass presented low T1 signal intensity and heterogeneously high T2 signal intensity. The uptake of gadolinium contrast was progressive, late and inhomogeneous. The morphological features, including enhancement pattern and lack of restriction on DW images, were those typical of a probably benign lesion.

Solitary neurofibromas are usually treated with surgical excision. Deep-seated lesions and lesions involving major nerve trunks are preferably treated conservatively, given that surgery requires sacrifice of the patient’s nerve.

Prognosis for solitary neurofibromas is usually excellent. Malignant degeneration is rare when not associated with NF-1 (there is a 4% chance of malignant degeneration in patients with NF-1) [7]. Local recurrence after excision is uncommon for lesions and not associated with NF-1. Major differentials include fibroadenoma and phylloides tumour of the breast.

Phylloides tumour is locally invasive with a high chance of recurrence after resection. The US appearance of phyllodes tumours is that of an inhomogeneous solid mass containing single or multiple round or cleft-like cystic areas. Posterior acoustic enhancement is sometimes present as is the case in some neurofibromas. Vascularisation is present within the solid component. Contrast-enhancement can either be gradual slow or rapid [10, 11].

Fibroadenoma is a common benign tumour of the breast resulting from excessive proliferation of connective tissue with minimal or no malignant potential. Fibroadenoma contains both stromal and epithelial cells. On US, the most common appearance is that of homogeneous hypoechoic well-circumscribed, round or oval nodule. Margins can be smooth or macrolobulated. The enhancement pattern on breast MR is similar to that of neurofibroma [12, 13].

## Conclusion

Solitary neurofibroma of the breast is an uncommon benign tumour, which can mimic other more aggressive tumours. Diagnosis relies on pathology; however, imaging plays an important role. MRI is the best imaging modality to evaluate morphology and enhancement pattern suggestive of a benign lesion.
